# Associations of Various Nighttime Noise Exposure Indicators with Objective Sleep Efficiency and Self-Reported Sleep Quality: A Field Study

**DOI:** 10.3390/ijerph16203790

**Published:** 2019-10-09

**Authors:** Martin Röösli, Mark Brink, Franziska Rudzik, Christian Cajochen, Martina S. Ragettli, Benjamin Flückiger, Reto Pieren, Danielle Vienneau, Jean-Marc Wunderli

**Affiliations:** 1 Swiss Tropical and Public Health Institute, 4051 Basel, Switzerland; Martina.ragettli@swisstph.ch (M.S.R.); benjamin.flueckiger@swisstph.ch (B.F.); danielle.vienneau@swisstph.ch (D.V.); 2 University of Basel, 4003 Basel, Switzerland; 3 Federal Office for the Environment, 3003 Bern, Switzerland; Mark.Brink@bafu.admin.ch; 4 Centre for Chronobiology, Psychiatric Hospital of the University of Basel, 4002 Basel, Switzerland; franziska.rudzik@posteo.de (F.R.); Christian.Cajochen@upk.ch (C.C.); 5 Transfaculty Research Platform Molecular and Cognitive Neurosciences, University of Basel, 4003 Basel, Switzerland; 6 Empa, Laboratory for Acoustics/Noise Control, Swiss Federal Laboratories for Materials Science and Technology, 8600 Dübendorf, Switzerland; Reto.Pieren@empa.ch (R.P.); Jean-Marc.Wunderli@empa.ch (J.-M.W.)

**Keywords:** sleep quality, road traffic noise, actimetry, indoor noise, noise measurements, noise annoyance, noise sensitivity, time of day

## Abstract

It is unclear which noise exposure time window and noise characteristics during nighttime are most detrimental for sleep quality in real-life settings. We conducted a field study with 105 volunteers wearing a wrist actimeter to record their sleep during seven days, together with concurrent outdoor noise measurements at their bedroom window. Actimetry-recorded sleep latency increased by 5.6 min (95% confidence interval (CI): 1.6 to 9.6 min) per 10 dB(A) increase in noise exposure during the first hour after bedtime. Actimetry-assessed sleep efficiency was significantly reduced by 2%–3% per 10 dB(A) increase in measured outdoor noise (L_eq, 1h_) for the last three hours of sleep. For self-reported sleepiness, noise exposure during the last hour prior to wake-up was most crucial, with an increase in the sleepiness score of 0.31 units (95% CI: 0.08 to 0.54) per 10 dB(A) L_eq,1h_. Associations for estimated indoor noise were not more pronounced than for outdoor noise. Taking noise events into consideration in addition to equivalent sound pressure levels (L_eq_) only marginally improved the statistical models. Our study provides evidence that matching the nighttime noise exposure time window to the individual’s diurnal sleep–wake pattern results in a better estimate of detrimental nighttime noise effects on sleep. We found that noise exposure at the beginning and the end of the sleep is most crucial for sleep quality.

## 1. Introduction

There is increasing epidemiological research demonstrating the negative effects of transportation noise exposure on various chronic diseases, such as cardiovascular disease [1,2,3,4], metabolic syndrome [5,6,7,8,9], depression [10,11,12], and cognitive functions [13,14,15,16]. Several mechanism are implicated in these negative noise effects, such as activation of the hypothalamic–pituitary–adrenal axis (HPA), which leads to increased cortisol and glucose levels as well as increased blood pressure, with consequences for blood viscosity and blood coagulation [17]. Also, chronic sleep deprivation is a stressor, which contributes to allostatic load and has been found to be connected to all these outcomes above. High allostatic load, characterized by repeated stress responses, affects the brain regions involved in memory consolidation and affective processing, with potential long-term effects on cognitive and mental health [18]. In large population studies, impaired sleep quantity and quality has been associated with increased risk of developing coronary heart diseases [19]. There is also solid evidence that short sleep duration, lack of slow-wave sleep, and circadian desynchronization of sleep increases sensitivity to food stimuli, contributing to adverse metabolic traits, in particular obesity and type 2 diabetes [20]. Further, sleep deprivation reduces the motivation for physical activity and thus reduces the energy expenditure [21], which further contributes to the risk of cardiometabolic syndromes.

Noise-induced sleep disturbance, as demonstrated in experimental human laboratory studies [22,23,24], field trials [25], and observational epidemiological studies [26,27], are thus likely to be on the pathway for detrimental effects on the cardiometabolic system and mental health. Strikingly, evidence for noise effects on various sleep outcomes was only considered “very low” to “moderate” in the recent systematic review of the World Health Organization Environmental Noise Guidelines for the European region [28]. Thus, many questions remain open. 

Sound pressure level at the ear of the sleeper is often regarded as the only relevant entity for quantifying noise effects on sleep. It is possible to quantify reactions of sleepers to sound pressure level at the ear in field experiments using contrived exposure settings (i.e., reproducing noise in a controlled fashion with loudspeakers). This approach, however, does not adequately consider long-term habituation to a noise source, which may be relevant in a real-life situation at home. Observational studies would be appropriate to account for potential habituation effects, but they are usually based on noise exposure modeling for the outdoor façade and are thus rather imprecise regarding noise exposure at the ear.

Further, the average sound pressure level (L_eq_ and similar indicators) may not be the only relevant factor; other noise exposure characteristics not captured in energy-based exposure indicators may also be important. For instance, in experimental sleep studies of noise effects on sleep, different effects have been observed for road, rail, and aircraft noise [22,23]. Thus, the effects of noise on sleep might be better predicted by the number of noise events [29], the maximum sound pressure level [30], the sound pressure level slope [24,31], or the order of events [31]. Confronted with the challenge of how to sum up and weight noise events, we developed an acoustical metric, the intermittency ratio (IR), to characterize short-term temporal variations of transportation noise exposure [32]. In a large cohort study, we found some evidence that IR may have a modifying effect on the cardiovascular mortality risk [33]. 

Timing of noise exposure is also considered to be relevant for sleep effects. For instance, an experimental sleep study observed that noise curfews at the end of the night were most beneficial for sleep because noise-induced sleep disturbances at the beginning of the night were at least partly compensated during the rest of the night [34].

In order to quantify relevant factors that affect sleep quality through noise exposure in a real-life situation, we conducted an observational field study with volunteers wearing wrist actimeters to record their sleep–wake behavior during seven days with concurrent indoor and outdoor noise exposure measurements. The study explored (i) the relevance of indoor noise compared to outdoor noise, (ii) the predictive contribution of IR to sleep effects in addition to equivalent continuous sound pressure levels (L_eq,night_), and (iii) the effect of noise exposure at different times during the night. We also tested potential effect modification by noise annoyance, noise sensitivity, and sex.

## 2. Materials and Methods 

### 2.1. Study Population and Procedures

Study participants were recruited from a previous nationwide survey on transportation noise annoyance and sleep disturbances who expressed their willingness to be contacted for further research [35]. Only adults with German language skills were included. People with severe chronic disease or shift work were excluded. Calculated L_den_ for road traffic at the most exposed façade had to be at least 50 dB to ensure sufficient outdoor noise to be detected inside. In order to explore the effects of road traffic noise exposure minimally contaminated by other noise sources, modeled aircraft and railway noise had to be at least 10 dB lower than road traffic noise. During recruitment, we ensured that no other relevant noise sources, such as construction work or tramways, could disturb the sound level measurements.

After recruitment, study participants were visited at home between May and November 2016 and instructed about the procedures. All participants filled in a baseline questionnaire about sociodemographic and other relevant characteristics, such as noise annoyance (using the 11-point ICBEN scale for road traffic noise in general [36]), noise sensitivity (using a 6-point Likert scale to rate the statement “I am noise-sensitive” from NoiSeQ) [37], and window opening habits during night. 

### 2.2. Sleep Outcomes

Objective sleep behavior was evaluated using movement data collected by an actimeter device (Daqtometer v2.4 by Daqtix GbR, Oetzen, Germany) continuously worn on the nondominant wrist during the seven-day study period. Data were collected in 10 to 60 s bins. Participants were provided with diaries in which they logged sleep-relevant information two times during the day. Prior to sleep, they gave information on consumption of coffee and alcohol, daytime naps, screen time use, and medication intake during the preceding day. In the mornings, participants evaluated the preceding sleep episode and noted bed and rise times and whether or not they used an alarm clock. Additionally, self-reported sleep quality was rated on a verbally anchored visual analogue scale from 0 (the worst sleep) to 100 (the best sleep). Self-reported sleepiness was rated on a verbally anchored Likert-type scale that ranged from 1 (extremely alert) to 9 (very sleepy–fighting sleep) [38].

Actimetry data were analyzed using the "Actiwatch Activity and Sleep Analysis" software (Actiwatch software version 7, Cambridge Neurotechnology Ltd., Cambridge, UK). Bed and rise times were entered manually based on actimetry records and sleep diary entries, while sleep on- and offset were determined automatically by the algorithm to derive sleep efficiency (proportion of actual sleep time per time in bed), sleep duration, sleep latency (time between bedtime and sleep onset), and moving time (minutes moving per assumed sleep period as time between sleep onset and offset).

### 2.3. Noise Measurements and Modeling

Noise exposure assessment included a seven-day outdoor measurement, a controlled short-term measurement to derive the sound insulation of each bedroom for different window positions, and noise exposure modeling to identify eligible households. We modeled road traffic noise exposure calculation at the most exposed façade of each study participant using the sonROAD emission model [39] and the sound propagation model StL-86, as described in detail in Karipidis et al., 2014 [40]. 

Outdoor noise at the study participant’s bedroom window was measured with sound level meter type Noise-Sentry RT, a class II measurement device with a measurement uncertainty of about 1 dB(A) [41]. The sound level meters were flush-mounted to the outer face of the closed window and logged A-weighted 1-second-L_eq_’s. To obtain free field estimates, a correction of −6 dB was applied to the raw data. After a measurement period of seven days, the participants removed the sound level meters and sent them back. From the measurement files, L_eq,night_ and IR_night_ (23:00–7:00) were computed for each night and participant. IR was calculated based on its definition [32] with a 1 h evaluation interval for the event-related sound exposure, *L*_eq,1h,Events_ and the total sound pressure value L_eq,1h,tot_. Every noise recording that exceeded L_eq,1h.tot_ by 3 dB was considered to be an event and contributed to the L_eq,1h,Events_. L_eq_ and IR for a priori specified time windows during the night were derived: 19:00–23:00, 23:00–1:00, 1:00–5:00, 5:00–6:00, and 6:00–7:00. We also calculated, for each individual, L_eq,1h_ exposure for the first four hours of sleep as well as the last four hours prior to wake-up, taking into account their individual sleep pattern on the corresponding night.

$$IR=\frac{10^{L_{\mathrm{eq},1h,\mathrm{Events}}/10}}{10^{L_{\mathrm{eq},1h,\mathrm{tot}}/10}}\cdot 100$$

Sound transmission of outdoor noise into the bedroom was measured during the home visit. Outdoor and indoor (at the position of the pillow) levels were measured in parallel for three window positions (open, tilted, closed) during three minutes in one-third octave bands from 50 Hz to 10 kHz with a class I sound level meter (type NTI XL2, NTi Audio AG, Schaan, Liechtenstein) and a free field microphone. Temporal resolution was set to one second. The indoor measurements were compared with the concurrently conducted outdoor measurements, and an algorithm was developed to derive the A-weighted attenuation factor in dB based on the correlation and the offset of these parallel measurements [42]. Finally, estimated indoor levels for analysis were obtained for each study participant by subtracting the individual attenuation factor from the outdoor measurements, taking into account the preferred window position for the season when the data were collected, as indicated in the baseline questionnaire.

### 2.4. Data Analysis

Primary endpoints were actimeter-derived sleep efficiency, sleep latency, sleep duration, and moving time. Secondary endpoints were self-reported sleep quality and sleepiness, as rated each morning in the sleep diary.

Association between sleep outcomes and noise exposure was analyzed using mixed regression models with auto-correlated residuals lag = 1 and robust standard errors. To account for repeated nights from the same individual and multiple study participation per household, a three-level random intercept model was applied. Models were adjusted for confounding factors, as indicated in the footnote of corresponding result tables (Tables 3 and 4). Effect modification by noise annoyance and noise sensitivity was investigated by interaction analysis on dichotomized variables.

## 3. Results

For the study, we included 107 individuals from 96 households, resulting in 720 nights with complete noise exposure data and recorded data on at least one sleep outcome (actimeter-derived or self-reported). Data from two individuals (14 nights) were excluded because their sleep was affected by their children. An additional 10 nights were excluded from the dataset because of sleeping out of home, two nights due to acute respiratory infection, and nine nights due to a recorded sleep duration of less than four hours. This left 694 nights from 105 individuals from 94 households for further analyses, although the number of observations were somewhat smaller for specific outcomes because actimetry data was not available for two individuals and self-reported sleep quality data was not available for two individuals.

The mean age of the study participants was 52.1 years (SD = 14.4 years, age range: 23 to 78 years). Fifty-three participants (51%) were female and 52 were male. Twenty-nine individuals (28%) had a university degree, 34 (32%) had a higher education, 39 (37%) had an apprenticeship, and 3 (3%) had compulsory education. Median noise annoyance was 6, with 23% being classified as highly annoyed (score ≥8). Forty-one subjects (39%) tended to agree (score 4–6) to the statement “I am noise-sensitive”.

Average sleep efficiency as recorded by actimetry was 88%, and average sleep duration was 7.0 h (Table 1). Self-reported sleep quality scores ranged from 4 to 100, with a mean of 65. Average self-reported sleepiness score was 4.1. Sleep efficiency was strongly negatively correlated with sleep latency (−0.79), and self-reported sleep quality was negatively correlated with self-reported sleepiness (−0.49). Correlations of all other outcomes were low (see Supplementary Table S1). 

Table 2 shows the summary estimates for the various noise exposure metrics. Mean measured nighttime noise level (L_eq,night_) outside the window of the study participants’ bedroom was 47.0 dB(A), with a maximum of 62.7 dB(A). Estimated mean indoor nighttime noise level at the pillow of the study participants was 30.2 dB(A) (maximum: 55.3 dB(A)). Measured exposure was about 10 dB(A) lower during 01:00 to 05:00 compared to the beginning and end of the night. Correlation between estimated indoor and measured outdoor nighttime exposure was 0.46, reflecting variation in noise attenuation between study participants. Correlations were ≥0.63 for measured L_eq_ between different time periods of the night (see Supplementary Table S3). 

Measured nighttime noise (L_eq,night_) tended to be negatively associated with sleep efficiency and positively associated with sleep latency, although they were not statistically significant (Table 3). For instance, sleep efficiency decreased by 1.11% (95% confidence interval (CI): −2.44% to 0.21%) and sleep latency increased by 5.67 min (95% CI: −1.00 to 12.34) per 10 dB(A) increase in L_eq,night_. No indications of an association (*p* ≥ 0.27) were seen for the other sleep outcomes, including self-reported sleep quality and sleepiness. This association pattern was also found in the raw data (Figure S1).

Using estimated indoor noise instead of measured outdoor noise yielded similar results in terms of regression coefficients, but none of the associations was even close to significance (Table 4). Similarly, we did not obtain any indications that IR was associated with any of the objectively recorded or self-reported sleep outcomes when also considering L_eq,night_ in the same model (see Supplementary Table S4). A model with quartiles of IR_night_ instead of a continuous variable suggested a nonlinear association for sleep efficiency and latency. For the second quartile of IR_night_ (50%–63%), sleep efficiency was reduced by 1.24% (95% CI: −2.73 to 0.25), and sleep latency was increased by 3.48 min (95% CI: −2.54 to 9.50) compared to the first quartile (4%–50%).

**Figure 1:**
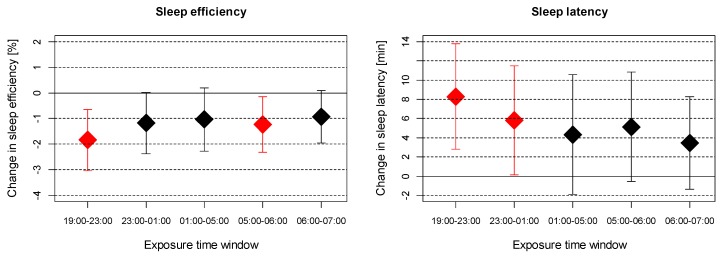
Association between measured outdoor noise exposure (L_eq_) at different time windows during night and changes in all-night sleep efficiency and sleep latency per 10 dB(A) increase in noise exposure. Significant associations are highlighted in red. Analyses adjusted for age, sex, education, evening caffeine intake, evening alcohol consumption, evening screen time, day of the week, season, and whether woken up by an alarm clock.

As most indications for noise effects were found for sleep efficiency and sleep latency in relation to outdoor noise, we investigated the effect of noise exposure in potential critical time windows for these two outcomes in more detail (Figure 1). Noise exposure in the evening (19:00–23:00) and in the early morning hours (05:00–06:00) was significantly associated with sleep efficiency, whereas the other time windows reached only borderline significance. For sleep latency, noise exposure until 01:00 was most relevant. 

**Figure 2:**
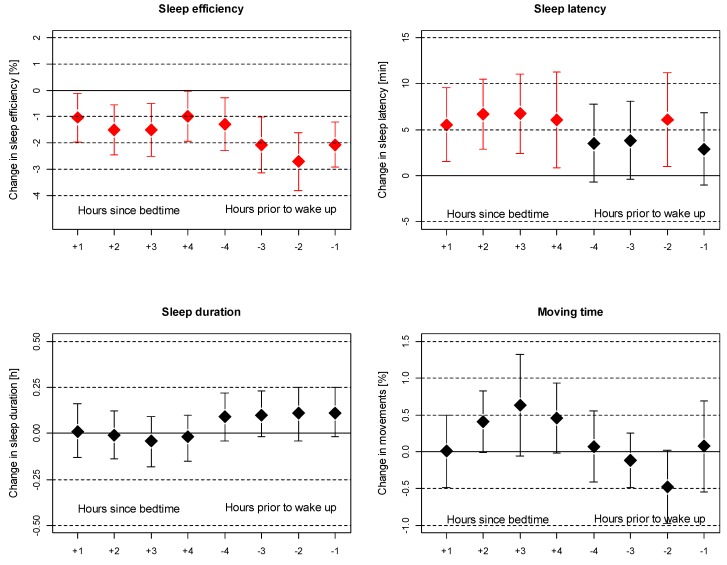
Association between actimetry-derived outcomes and outdoor noise exposure for each hour after bedtime (+) or noise exposure for each hour prior to wake-up (−). Changes refer to a 10 dB(A) increase in noise exposure in the respective hour. Same adjustments as indicated in Figure 1.

Exposure within fixed time periods (as shown above) may not match the individual time period a person is asleep or in bed. For instance, for somebody rising before 06:00, noise exposure between 06:00 and 07:00 is not relevant regarding sleep disturbance. We thus calculated hourly noise exposure levels that matched the individual sleeping pattern of each night for the first four and the last four hours of sleep. The corresponding analyses with all actimetry-derived sleep outcomes are shown in Figure 2. This individualized noise exposure provided stronger associations between measured noise exposure and sleep efficiency. Sleep latency increased by five to seven minutes per 10 dB(A) increase in outdoor noise exposure during the first four hours after bedtime. For sleep efficiency, noise exposure during the last three hours prior to wake-up was most critical, with reductions of 2%–3% per 10 dB(A) increase in measured outdoor noise exposure (L_eq,1h_). For sleep duration and moving time, no significant associations were found, although noise exposure two to four hours after bedtime tended to increase moving time during sleep, and noise exposure two hours before wake-up tended to be negatively associated with moving time.

**Figure 3:**
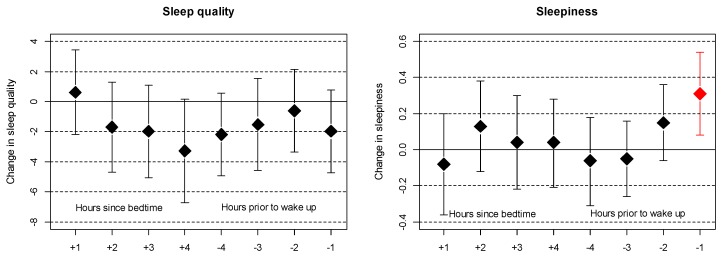
Association between self-reported outcomes recorded each morning in the sleep diary and outdoor noise exposure for each hour after bedtime (+) or noise exposure for each hour prior to wake-up (−). Changes refer to a 10 dB(A) increase in noise exposure in the respective hour. Same adjustments as indicated in Figure 1.

Self-reported sleepiness in the morning was significantly associated with noise exposure in the last hour of sleep, whereas no associations were observed for noise exposure at the beginning of sleep (Figure 3). For self-reported sleep quality, none of the noise exposure time windows were significantly associated, but noise exposure in the middle of the sleeping period (±4 h from bedtime and wake-up) and at the end of the sleeping period showed the strongest trends for a relation. For IR matched to individualized sleep patterns, no significant effects on actimetry-derived and self-reported sleep outcomes were observed.

Noise annoyance, noise sensitivity, and sex were not significant effect modifiers for any of the outcomes and noise exposure time windows. A slight, nonsignificant trend was seen for a stronger association of nighttime noise (L_eq,night_) with sleep efficiency for males (*p* = 0.11), people with a high (≥median) annoyance score (*p* = 0.23), and people not reporting to be noise-sensitive (*p* = 0.25).

## 4. Discussion

Our study suggests that the timing of noise exposure within the night is a relevant factor for the deterioration of objective and self-reported sleep quality. Using individual noise exposure time windows, matched to the individual bed and rise time of each night, provided stronger associations compared to fixed time intervals (such as L_eq_ between 19:00 and 23:00). Sleep latency, as expected, was most consistently associated with noise exposure at the beginning of the night, while noise exposure prior to wake-up was most relevant for sleep efficiency and self-rated sleepiness. This suggests that noise exposure in the middle of the night may be less relevant for sleep quantity and quality, whereas noise exposure toward the end of the night, when sleep pressure is reduced and noise levels tend to be higher, is most disturbing for sleep.

This is in line with a field study using contrived aircraft noise exposure observing that aircraft noise events in the early morning elicited stronger reactions, as measured with high-resolution actigraphy, than events at the beginning of the sleep period [31]. Our findings are also in line with a large population-based survey conducted in 2009–2010 in Oslo with 13,019 participants. Road traffic nighttime noise was mostly associated with waking up too early and with difficulties falling asleep but barely associated with awakenings during night [27]. In a Finnish study of 7019 public sector employees, people exposed to >55 dB road traffic nighttime noise were more likely to report nonrestorative sleep (odds ratio (OR) = 1.29, 95% CI: 1.01–1.65) and waking up too early (OR = 1.24, 95% CI: 0.96–1.61) compared to people exposed to 45 dB or less. No associations were observed for frequently waking up during the night, short sleep duration, and difficulties falling asleep, with the latter not in line with our results [43]. In a smaller Swiss survey of 1375 adults, various self-reported sleep quality indices were not significantly related to road traffic nighttime noise [26]. However, most indications for an exposure–response trend were found for “waking up too early in the morning”, while least indications were found for “agitated sleep” and “waking phases during the night” in that same study [26]. 

Whether, or how, the observed pattern with stronger effects for evening and early morning noise translates into chronic health effects is unclear. Separating long-term noise effects regressed on average night noise exposure from time-specific effects in specific time periods is challenging for epidemiological studies, primarily because transportation noise in different time periods within the night is highly correlated, at least if no night curfews are in force. This is especially the case for modeled road traffic noise when traffic input data are based on traffic count samples, which are then extrapolated [2,3]. In reality, as demonstrated in our study, diurnal and day-to-day variation in traffic flows leads to a lower correlation between environmental noise exposures at different time intervals than one would observe between such computed standard metrics [44]. For large-scale epidemiological studies on long-term risks, individual noise measurements, as done here, are not feasible. A Swiss cohort study on cardiovascular mortality evaluated the effects of diurnal noise variation in their analysis by combining road, rail, and aircraft noise. Although such a combination introduces additional diurnal variability due to different pattern and night curfews for aircraft noise, the correlation between different exposure time windows remained high (≥0.94), precluding any firm conclusion. There was a trend that, for all cardiovascular causes combined, exposure during 01:00 to 05:00 was most relevant. For hypertensive-related causes of death, early morning noise (05:00–06:00) was most relevant, and for ischemic stroke and heart failure, early evening and early morning noise were more detrimental than the rest of the night, although strongest association were seen with daytime noise [45]. 

We hypothesized that we would find stronger associations for estimated indoor noise than measured outdoor noise, but we could not confirm this with our data. This is in line with results from a survey on self-reported sleep disturbance in the same Special Issue of this journal [46]. However, our findings do not match the results of an epidemiological study done on indoor noise, which found stronger associations for hypertension with indoor noise compared to modeled noise at the most exposed façade [47]. 

We did not measure indoor noise but rather applied an indirect procedure to estimate indoor noise levels from measured outdoor noise because our main interest was outdoor noise penetrating into the building. Direct indoor noise measurements would be heavily affected by the behavior of the participants. For instance, a sleepless person may produce some sound, which would yield a biased correlation between sleeplessness and noise exposure. There is no obvious explanation why indoor noise was less good a predictor than outdoor noise in our study. One may speculate that people who feel disturbed by outdoor noise close their bedroom window and thus have a lower indoor noise estimate, as this is the strongest predictor of indoor noise. This would mean that we deal with reverse causation in the sense that the sleep quality affects the noise exposure and not the other way round. Alternatively, estimated indoor noise levels may be subject to higher exposure misclassification as some levels were low. Estimated indoor noise levels below 20 dB(A) were censored and replaced with 20 dB(A) for the analysis; this was the case for 79 nights in 21 individuals. Finally, we obtained window opening habits for each season from the baseline questionnaire but did not specifically ask about the window position for each night, which may also add to exposure misclassification. Exposure misclassification may have resulted in reduced statistical power, which would explain the observed similar regression coefficients for indoor and outdoor noise but higher *p*-values for indoor noise.

We also hypothesized that sleep effects depend on the exposure characteristics. In particular, exposure situations with individual noise events clearly standing out from average (background) noise, as quantified with the IR [32], were considered to be more detrimental for sleep than exposure to a steady sound level. However, we could not confirm that sleep disturbances are increasing with increasing IR. There was a nonsignificant, nonlinear pattern with lowest sleep efficiency for moderate (50%–63%) levels of IR, which is in line with findings of the effect of IR in a cohort study on cardiovascular mortality [33]. However, in a cross-sectional survey on arterial stiffness, number of events during night was relevant [48], and in a cohort study on diabetes, IR was not associated with the diabetes risk [7]. Thus, it remains open whether IR is contributing to L_eq_-based metrics for predicting long-term health effects of noise.

The strengths of this study include the prospective and detailed data collection with acquisition of objective sleep data (actimetry) and measurement of noise exposure. By measuring outdoor noise exposure instead of calculating it, we could adequately record the diurnal variability of noise acting upon study participants. Our measurements allowed us to estimate outdoor noise passing into the sleeping rooms for individual bedroom characteristics. We could also match exposure time windows to the individual bed and rise times. The relatively small sample is a limitation, and thus the power of the study is rather limited. The *p*-values of the analyses are not adjusted for multiple comparisons because we were interested in the pattern of the effect estimates rather than hypothesis testing. Thus, some significant coefficients may, in fact, be chance findings. Note also that some of the exposure and outcome measures were correlated, and thus analysis should not be considered as mutually independent. For instance, sleep latency was found to be significantly associated with noise exposure two hours prior to wake-up. This seemingly paradoxical result is likely explained by the fact that noise exposure in the early morning is correlated with noise exposure noise late at night. Only the latter is causally related to sleep latency.

## 5. Conclusions

Our study provides evidence that matching the nighttime noise exposure time window to the individual’s diurnal sleep–wake pattern results in a better estimate of detrimental nighttime noise effects on sleep. The study suggests that noise exposure in the early morning hours is probably most crucial for a negative impact on objective sleep efficiency and self-reported sleep quality. However, evening noise exposure was also associated with longer sleep latency. We could not confirm that noise-induced sleep effects are better explained by indoor noise compared to outdoor noise, which might be due to reverse causality. This needs to be confirmed in larger studies.

## Figures and Tables

**Table 1:** Overview of the sleep outcomes (ACT—actimetry; SR—self-reported).

Outcome	N	Mean	SD	Min	Max
ACT sleep efficiency [%]	634	88.4	7.9	46.3	98.2
ACT sleep latency [min]	634	29.4	33	2	303
ACT sleep duration [h]	634	7.0	1.2	4.1	11.7
ACT moving time [%]	634	7.2	4.7	0.8	35.9
SR sleep quality [0–100]	639	65	20	4	100
SR sleepiness [1–9]	633	4.1	1.8	1	9

**Table 2:** Overview of the noise exposure data in dB(A) (for intermittency ratio (IR), see Supplementary Table S2).

Outcome	N	Mean	SD	Min	Max
Indoor L_eq,night_	685	30.2	7.6	20.0	55.3
Outdoor L_eq,night_	685	47.0	6.9	29.6	62.7
Outdoor L_eq,19–23_	685	51.2	6.6	33.2	68.6
Outdoor L_eq,23–01_	685	46.1	7.4	29.4	62.9
Outdoor L_eq,01–05_	685	41.8	7.5	27.8	62.7
Outdoor L_eq,05–06_	685	46.3	8.1	28.8	64.7
Outdoor L_eq,06–07_	685	50.6	8.1	29.5	70.3

Note: All estimated indoor values <20 dB(A) have been replaced by 20 dB(A).

**Table 3:** Adjusted association between sleep outcomes and measured outdoor nighttime noise (L_eq,night_) per 10 dB(A) increase.

Outcome	N	Coefficient	Confidence Interval	*p*-Value
ACT sleep efficiency [%]	634	−1.11	−2.44 to 0.21	0.10
ACT sleep latency [min]	634	5.67	−1.00 to 12.34	0.10
ACT sleep duration [h]	634	0.01	−0.17 to 0.19	0.94
ACT moving time [%]	634	−0.41	−1.13 to 0.31	0.27
SR sleep quality [0–100]	639	−1.09	−4.96 to 2.78	0.58
SR sleepiness [1–9]	633	0.02	−0.29 to 0.32	0.91

Note: Adjusted for age, sex, education, evening caffeine intake, evening alcohol consumption, evening screen time, day of the week, season, and whether woken up by an alarm clock.

**Table 4:** Adjusted association between sleep outcomes and estimated indoor nighttime noise (L_eq,night_) per 10 dB(A) increase.

Outcome	N	Coefficient	Confidence Interval	*p*-Value
ACT sleep efficiency [%]	634	−1.06	−2.86 to 0.74	0.25
ACT sleep latency [min]	634	4.39	−5.54 to 14.32	0.39
ACT sleep duration [h]	634	−0.06	−0.21 to 0.10	0.48
ACT moving time [%]	634	−0.24	−0.90 to 0.42	0.47
SR sleep quality [0–100]	639	0.21	−3.46 to 3.88	0.91
SR sleepiness [1–9]	633	−0.01	−0.28 to 0.26	0.95

Note: Adjusted for age, sex, education, evening caffeine intake, evening alcohol consumption, evening screen time, day of the week, season, and whether woken up by an alarm clock.

## Supplementary Materials

The following are available online at https://www.mdpi.com/1660-4601/16/20/3790/s1, Table S1: Pearson correlation matrix of sleep outcomes. Table S2: Summary of IR exposure data, Table S3: Pearson correlation matrix for measured nighttime noise exposure metrics, Figure S1: Scatter plots of raw outcome data in relation to nighttime outdoor noise exposure, Table S4: Association between all outcomes and IR_night_.

## References

[1] Heritier H., Vienneau D., Foraster M., Eze I.C., Schaffner E., de Hoogh K., Thiesse L., Rudzik F., Habermacher M., Kopfli M. (2019). A systematic analysis of mutual effects of transportation noise and air pollution exposure on myocardial infarction mortality: A nationwide cohort study in Switzerland. Eur. Heart J..

[2] Sorensen M., Andersen Z.J., Nordsborg R.B., Jensen S.S., Lillelund K.G., Beelen R., Schmidt E.B., Tjonneland A., Overvad K., Raaschou-Nielsen O. (2012). Road traffic noise and incident myocardial infarction: A prospective cohort study. PLoS ONE.

[3] Cai Y., Hodgson S., Blangiardo M., Gulliver J., Morley D., Fecht D., Vienneau D., de Hoogh K., Key T., Hveem K. (2018). Road traffic noise, air pollution and incident cardiovascular disease: A joint analysis of the hunt, epic-oxford and uk biobank cohorts. Environ. Int..

[4] Hansell A.L., Blangiardo M., Fortunato L., Floud S., de Hoogh K., Fecht D., Ghosh R.E., Laszlo H.E., Pearson C., Beale L. (2013). Aircraft noise and cardiovascular disease near heathrow airport in London: Small area study. BMJ.

[5] Foraster M., Eze I.C., Vienneau D., Schaffner E., Jeong A., Heritier H., Rudzik F., Thiesse L., Pieren R., Brink M. (2018). Long-term exposure to transportation noise and its association with adiposity markers and development of obesity. Environ. Int..

[6] Christensen J.S., Hjortebjerg D., Raaschou-Nielsen O., Ketzel M., Sorensen T.I., Sorensen M. (2016). Pregnancy and childhood exposure to residential traffic noise and overweight at 7years of age. Environ. Int..

[7] Eze I.C., Foraster M., Schaffner E., Vienneau D., Heritier H., Rudzik F., Thiesse L., Pieren R., Imboden M., von Eckardstein A. (2017). Long-term exposure to transportation noise and air pollution in relation to incident diabetes in the sapaldia study. Int. J. Epidemiol..

[8] Ohlwein S., Hennig F., Lucht S., Matthiessen C., Pundt N., Moebus S., Jockel K.H., Hoffmann B. (2019). Indoor and outdoor road traffic noise and incident diabetes mellitus: Results from a longitudinal german cohort study. Environ. Epidemiol..

[9] Sorensen M., Andersen Z.J., Nordsborg R.B., Becker T., Tjonneland A., Overvad K., Raaschou-Nielsen O. (2013). Long-term exposure to road traffic noise and incident diabetes: A cohort study. Environ. Health Perspect..

[10] Beutel M.E., Junger C., Klein E.M., Wild P., Lackner K., Blettner M., Binder H., Michal M., Wiltink J., Brahler E. (2016). Noise annoyance is associated with depression and anxiety in the general population—The contribution of aircraft noise. PLoS ONE.

[11] Orban E., McDonald K., Sutcliffe R., Hoffmann B., Fuks K.B., Dragano N., Viehmann A., Erbel R., Jockel K.H., Pundt N. (2016). Residential road traffic noise and high depressive symptoms after five years of follow-up: Results from the heinz nixdorf recall study. Environ. Health Perspect..

[12] Seidler A., Hegewald J., Seidler A.L., Schubert M., Wagner M., Droge P., Haufe E., Schmitt J., Swart E., Zeeb H. (2017). Association between aircraft, road and railway traffic noise and depression in a large case-control study based on secondary data. Environ. Res..

[13] Clark C., Crombie R., Head J., van Kamp I., van Kempen E., Stansfeld S.A. (2012). Does traffic-related air pollution explain associations of aircraft and road traffic noise exposure on children’s health and cognition? A secondary analysis of the United Kingdom sample from the ranch project. Am. J. Epidemiol..

[14] Stansfeld S.A., Berglund B., Clark C., Lopez-Barrio I., Fischer P., Ohrstrom E., Haines M.M., Head J., Hygge S., van Kamp I. (2005). Aircraft and road traffic noise and children’s cognition and health: A cross-national study. Lancet.

[15] Van Kempen E., van Kamp I., Lebret E., Lammers J., Emmen H., Stansfeld S. (2010). Neurobehavioral effects of transportation noise in primary schoolchildren: A cross-sectional study. Environ. Health.

[16] Sygna K., Aasvang G.M., Aamodt G., Oftedal B., Krog N.H. (2014). Road traffic noise, sleep and mental health. Environ. Res..

[17] Munzel T., Schmidt F.P., Steven S., Herzog J., Daiber A., Sorensen M. (2018). Environmental noise and the cardiovascular system. J. Am. Coll. Cardiol..

[18] McEwen B.S. (2006). Sleep deprivation as a neurobiologic and physiologic stressor: Allostasis and allostatic load. Metabolism.

[19] Cappuccio F.P., Cooper D., D’Elia L., Strazzullo P., Miller M.A. (2011). Sleep duration predicts cardiovascular outcomes: A systematic review and meta-analysis of prospective studies. Eur. Heart J..

[20] Schmid S.M., Hallschmid M., Schultes B. (2015). The metabolic burden of sleep loss. Lancet Diabetes Endocrinol..

[21] DePorter D.P., Coborn J.E., Teske J.A. (2017). Partial sleep deprivation reduces the efficacy of orexin-a to stimulate physical activity and energy expenditure. Obesity.

[22] Basner M., Muller U., Elmenhorst E.M. (2011). Single and combined effects of air, road, and rail traffic noise on sleep and recuperation. Sleep.

[23] Elmenhorst E.M., Griefahn B., Rolny V., Basner M. (2019). Comparing the effects of road, railway, and aircraft noise on sleep: Exposure(-)response relationships from pooled data of three laboratory studies. Int. J. Environ. Res. Public Health.

[24] Rudzik F., Thiesse L., Pieren R., Wunderli J.M., Brink M., Foraster M., Heritier H., Eze I.C., Garbazza C., Vienneau D. (2018). Sleep spindle characteristics and arousability from nighttime transportation noise exposure in healthy young and older individuals. Sleep.

[25] Elmenhorst E.M., Pennig S., Rolny V., Quehl J., Mueller U., Maass H., Basner M. (2012). Examining nocturnal railway noise and aircraft noise in the field: Sleep, psychomotor performance, and annoyance. Sci. Total Environ..

[26] Frei P., Mohler E., Röösli M. (2014). Effect of nocturnal road traffic noise exposure and annoyance on objective and subjective sleep quality. Int. J. Hyg. Environ. Health.

[27] Evandt J., Oftedal B., Hjertager Krog N., Nafstad P., Schwarze P.E., Marit Aasvang G. (2017). A population-based study on nighttime road traffic noise and insomnia. Sleep.

[28] Basner M., McGuire S. (2017). Who environmental noise guidelines for the european region: A systematic review on environmental noise and effects on sleep. Int. J. Environ. Res. Public Health.

[29] Janssen S.A., Centen M.R., Vos H., van Kamp I. (2014). The effect of the number of aircraft noise events on sleep quality. Appl. Acoust..

[30] Basner M., Samel A., Isermann U. (2006). Aircraft noise effects on sleep: Application of the results of a large polysomnographic field study. J. Acoust. Soc. Am..

[31] Brink M., Lercher P., Eisenmann A., Schierz C. (2008). Influence of slope of rise and event order of aircraft noise events on high resolution actimetry parameters. Somnologie.

[32] Wunderli J.M., Pieren R., Habermacher M., Vienneau D., Cajochen C., Probst-Hensch N., Röösli M., Brink M. (2016). Intermittency ratio: A metric reflecting short-term temporal variations of transportation noise exposure. J. Expo. Sci. Environ. Epidemiol..

[33] Heritier H., Vienneau D., Foraster M., Eze I.C., Schaffner E., Thiesse L., Rudzik F., Habermacher M., Kopfli M., Pieren R. (2017). Transportation noise exposure and cardiovascular mortality: A nationwide cohort study from switzerland. Eur. J. Epidemiol..

[34] Griefahn B., Marks A., Robens S. (2008). Experiments on the time frame of temporally limited traffic curfews to prevent noise induced sleep disturbances. Somnologie.

[35] Brink M., Schaffer B., Vienneau D., Foraster M., Pieren R., Eze I.C., Cajochen C., Probst-Hensch N., Röösli M., Wunderli J.M. (2019). A survey on exposure-response relationships for road, rail, and aircraft noise annoyance: Differences between continuous and intermittent noise. Environ. Int..

[36] Fields J.M., De Jong R.G., Gjestland T., Flindell I.H., Job R.F.S., Kurra S., Lercher P., Vallet M., Yano T., Guski R. (2001). Standardized general-purpose noise reaction questions for community noise surveys: Research and a recommendation. J. Sound Vib..

[37] Griefahn B., Marks A., Gjestland T., Preis A. (2007). Annoyance and Noise Sensitivity in Urban Areas.

[38] Akerstedt T., Gillberg M. (1990). Subjective and objective sleepiness in the active individual. Int. J. Neurosci..

[39] Heutschi K. (2004). Sonroad: New swiss road traffic noise model. Acta Acust. United Acust..

[40] Karipidis I., Vienneau D., Habermacher M., Köpflii M., Brink M., Probst-Hensch N., Röösli M., Wunderli J.-M. (2014). Reconstruction of historical noise exposure data for environmental epidemiology in switzerland within the sirene project. Noise Mapp..

[41] Schlatter F., Piquerez A., Habermacher M., Ragettli M.S., Röösli M., Brink M., Cajochen C., Probst-Hensch N., Foraster M., Wunderli J.-M. (2017). Validation of large scale noise exposure modeling by long-term measurements. Noise Mapp..

[42] Locher B., Piquerez A., Habermacher M., Ragettli M., Röösli M., Brink M., Cajochen C., Vienneau D., Foraster M., Müller U. (2018). Differences between outdoor and indoor sound levels for open, tilted, and closed windows. Int. J. Environ. Res. Public Health.

[43] Halonen J.I., Vahtera J., Stansfeld S., Yli-Tuomi T., Salo P., Pentti J., Kivimaki M., Lanki T. (2012). Associations between nighttime traffic noise and sleep: The finnish public sector study. Environ. Health Perspect..

[44] Brink M., Schaeffer B., Pieren R., Wunderli J.M. (2018). Conversion between noise exposure indicators leq(24h), l-day, l-evening, l-night, l-dn and l-den: Principles and practical guidance. Int. J. Hyg. Environ. Health.

[45] Heritier H., Vienneau D., Foraster M., Eze I.C., Schaffner E., Thiesse L., Ruzdik F., Habermacher M., Kopfli M., Pieren R. (2018). Diurnal variability of transportation noise exposure and cardiovascular mortality: A nationwide cohort study from switzerland. Int. J. Hyg. Environ. Health.

[46] Brink M., Schäffer B., Vienneau D., Pieren R., Foraster M., Eze I.C., Rudzik F., Thiesse L., Cajochen C., Probst-Hensch N. (2019). Self-reported sleep disturbance from road, rail and aircraft noise: Exposure-response relationships and effect modifiers in the swiss SiRENE study. Int. J. Environ. Res. Public Health.

[47] Foraster M., Kunzli N., Aguilera I., Rivera M., Agis D., Vila J., Bouso L., Deltell A., Marrugat J., Ramos R. (2014). High blood pressure and long-term exposure to indoor noise and air pollution from road traffic. Environ. Health Perspect..

[48] Foraster M., Eze I.C., Schaffner E., Vienneau D., Heritier H., Endes S., Rudzik F., Thiesse L., Pieren R., Schindler C. (2017). Exposure to road, railway, and aircraft noise and arterial stiffness in the sapaldia study: Annual average noise levels and temporal noise characteristics. Environ. Health Perspect..

