# Supplementary material for: Associations of Various Nighttime Noise Exposure Indicators with Objective Sleep Efficiency and Self-Reported Sleep Quality: A Field Study
Source: Int J Environ Res Public Health. 2019 Oct 9;16(20):3790. doi: 10.3390/ijerph16203790 (PMC6843841; doi:10.3390/ijerph16203790)
Supplement: Supplementary file 1 [file ijerph-16-03790-s001.pdf]

Supplementary

A panel study on various noise exposure measures on objective and self-reported sleep quality

Martin Röösli[1,2,*], Mark Brink[3], Franziska Rudzik[4], Christian Cajochen[5], Martina S. Ragettli[1,2], Benjamin Flückiger[1,2], Reto Pieren[6], Danielle Vienneau[1,2], Jean-Marc Wunderli[6]

Table S1: Pearson correlation matrix of sleep outcomes. Positive correlations are highlighted in green, negative correlations in orange

	AW sleep efficiency	AW sleep latency	AW sleep duration	AW moving time	SR sleep quality	SR sleepiness
AW sleep efficiency [%]	1.00					
AW sleep latency [min]	-0.79	1.00				
AW sleep duration [h]	0.26	-0.10	1.00			
AW moving time [%]	-0.12	0.07	0.10	1.00		
SR sleep quality [0-100]	0.25	-0.24	0.01	-0.20	1.00	
SR sleepiness [1-9]	-0.19	0.19	-0.01	0.07	-0.48	0.00

Table S2: Summary of IR exposure data

Exposure metric	N	Mean	SD	Min	Max
IR_{23-07}	685	60.2	21.1	0.0	97.4
IR_{19-23}	685	59.5	21.0	1.7	98.4
IR_{23-01}	685	61.0	24.2	0.0	99.1
IR_{01-05}	685	56.0	25.7	0.0	98.9
IR_{05-06}	685	54.7	23.6	0.0	95.4
IR_{06-07}	685	54.1	23.3	0.3	99.0

Table S3: Pearson correlation matrix for measured night time noise exposure metrics: Leq and IR at different time window. Positive correlations are highlighted in green.

	Leq 23-07	Leq 19-23	Leq 23-01	Leq 01-05	Leq 05-06	Leq 06-07	IR 23-07	IR 19-23	IR 23-01	IR 01-05	IR 05-06	IR 06-07
Leq 23-07	1											
Leq 19-23	0.81	1										
Leq 23-01	0.86	0.78	1									
Leq 01-05	0.86	0.72	0.79	1								
Leq 05-06	0.88	0.76	0.70	0.82	1							
Leq 06-07	0.89	0.72	0.63	0.70	0.87	1						
IR 23-07	0.19	0.16	0.16	-0.03	-0.04	0.17	1					
IR 19-23	-0.10	0.13	-0.15	-0.21	-0.15	-0.05	0.63	1				
IR 23-01	0.18	0.23	0.26	-0.02	0.06	0.14	0.77	0.57	1			
IR 01-05	0.45	0.44	0.38	0.42	0.40	0.43	0.59	0.35	0.64	1		
IR 05-06	0.30	0.32	0.22	0.22	0.34	0.35	0.61	0.47	0.59	0.69	1	
IR 06-07	0.11	0.06	0.00	-0.05	-0.06	0.21	0.81	0.56	0.45	0.37	0.54	1

Figure S1: Scatter plots of raw outcome data in relation to nighttime outdoor noise exposure ($_{Leq,night}$); ACT =Actiwatch; SR=Self reported

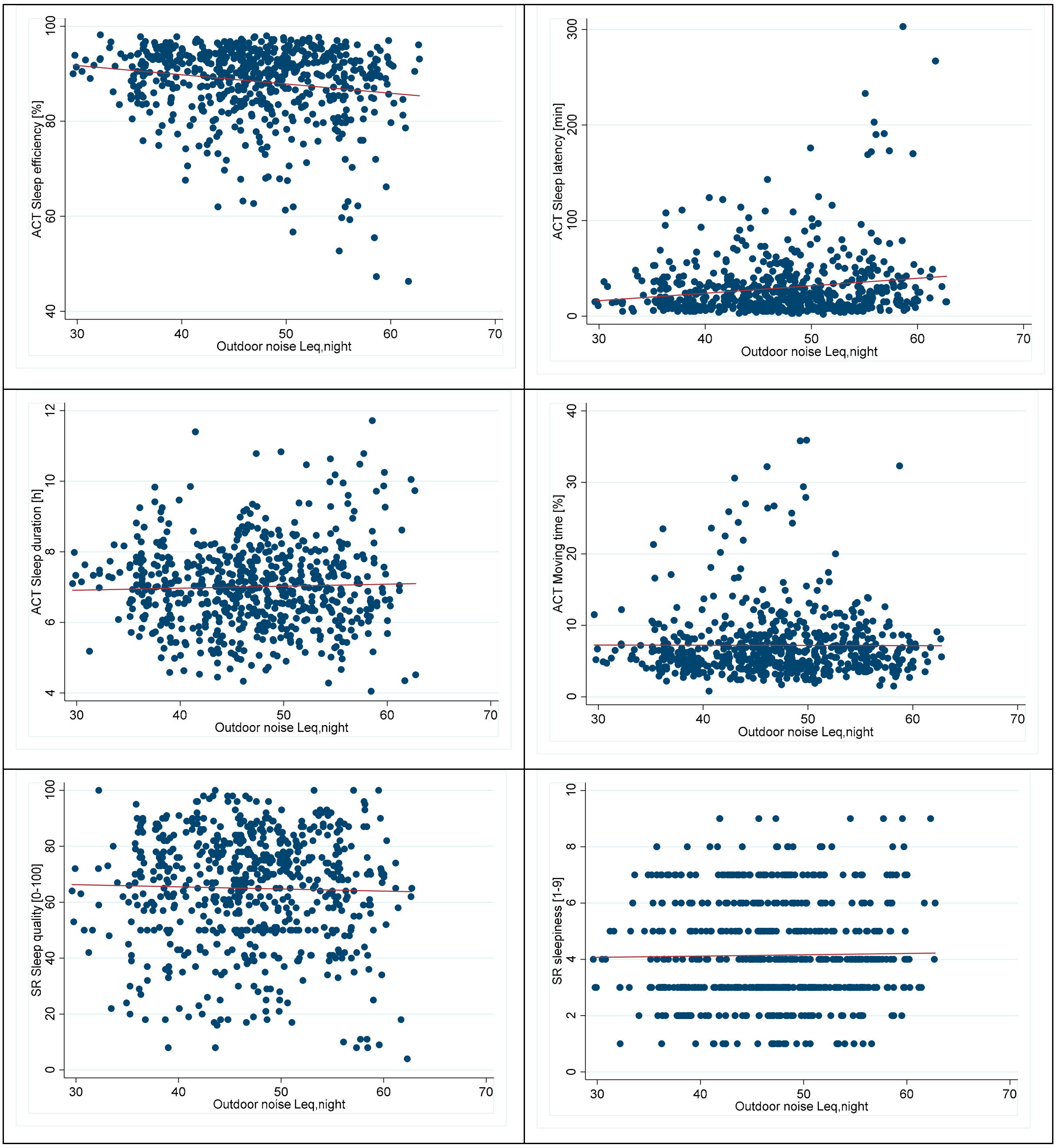

Table S4: Associations between all outcomes and IR_{night} calculated from the outdoor noise measurements per 10% increase in IR; ACT =Actiwatch; SR=Self reported

Outcome	N	Coefficient	Confidence interval	p-value
ACT Sleep efficiency [%]	634	0.02	-0.26 to 0.30	0.90
ACT Sleep latency [min]	634	-0.73	-1.86 to 0.39	0.20
ACT Sleep duration [h]	634	-0.03	-0.09 to 0.04	0.39
ACT Moving time [%]	634	-0.01	-0.17 to 0.15	0.89
SR Sleep quality [0-100]	639	-0.18	-1.11 to 0.76	0.71
SR sleepiness [1-9]	633	0.01	-0.07 to 0.09	0.79

[1] adjusted for $L_{eq,night}$, age, sex, education, evening caffeine intake, evening alcohol consumption, evening screen time, day of the week, season and whether woken up by an alarm clock
